# Algebraic Hyperstructures of Vague Soft Sets Associated with Hyperrings and Hyperideals

**DOI:** 10.1155/2015/780121

**Published:** 2015-07-12

**Authors:** Ganeshsree Selvachandran, Abdul Razak Salleh

**Affiliations:** ^1^Department of Actuarial Science and Applied Statistics, Faculty of Business and Information Science, UCSI University, Jalan Menara Gading, Cheras, 56000 Kuala Lumpur, Malaysia; ^2^School of Mathematical Sciences, Faculty of Science and Technology, Universiti Kebangsaan Malaysia (UKM), 43600 Bangi, Selangor, Malaysia

## Abstract

We apply the classical theory of hyperrings to vague soft sets to derive the concepts of vague soft hyperrings, vague soft hyperideals, and vague soft hyperring homomorphism. The properties and structural characteristics of these concepts are also studied and discussed. Furthermore, the relationship between the concepts introduced here and the corresponding concepts in classical hyperring theory and soft hyperring theory is studied and investigated.

## 1. Introduction

Hyperstructure theory which is a generalization of the notion of classical algebraic structures was introduced by Marty in 1934 (see [1]). Since its inception, hyperstructure theory has seen tremendous development. Corsini (see [2]) introduced the concept of a hyperring and gave the definition of the most general form of hyperring, besides being responsible for introducing the notion of hyperring homomorphism. In 1994, Vougiouklis (see [3]) introduced another type of hyperrings called the *H*_*ν*_-ring which is a generalization of the notion of hyperring introduced by [2]. Vougiouklis (see [3]) also introduced the notion of *H*_*ν*_-subring and left and right *H*_*ν*_-ideal of a *H*_*ν*_-ring.

The theory of hyperstructures has also been actively applied to various mathematical theories such as the theory of fuzzy sets introduced by Zadeh in 1965 (see [4]), intuitionistic fuzzy theory introduced by Atanassov in 1986 (see [5]), and soft set theory introduced by Molodtsov in 1999 (see [6]), to produce several important concepts and theories. The study of fuzzy algebra began with the introduction of the notion of a fuzzy subgroup of a group by Rosenfeld (see [7]). Consequently, this led to the study of fuzzy hyperalgebra beginning with the introduction of the notion of a fuzzy subhyperring of a hyperring (resp., fuzzy *H*_*ν*_-subrings) of a hyperring (resp., *H*_*ν*_-ring) and fuzzy hyperideals (resp., fuzzy *H*_*ν*_-ideals) of hyperrings (resp., *H*_*ν*_-rings). The notion of fuzzy hyperideals introduced by Davvaz (see [8]) is a generalization of the concept of Liu's (see [9]) definition of a fuzzy ideal of a ring.

Soft set theory is a general mathematical tool which was designed to deal with uncertainties and is widely acknowledged as an effective alternative to the classical mathematical tools that were used to deal with uncertainties and imprecision. Prior to the introduction of soft set theory, mathematical theories such as the theory of fuzzy sets, rough sets, and probability theory were used as tools to deal with uncertainties, imprecision, and vagueness. However, soft set theory has been proven to be a superior alternative to these classical theories as it is free from the inherent difficulties that affect these classical mathematical tools. Soft set theory has been successfully applied in various areas of mathematics such as in classical algebra and hyperalgebra, fuzzy algebra, and fuzzy hyperalgebra to develop various algebraic structures. However, an inherent weakness of soft set theory is that it is difficult to be used to represent the vagueness of problem parameters, particularly in problem-solving and decision making contexts. To overcome this disadvantage, various generalizations of soft sets such as fuzzy soft sets, vague soft sets, and intuitionistic fuzzy soft sets were introduced as better alternatives to the concept of soft sets. In the context of this paper, the notion of vague soft sets is used as a base to develop the initial theory of vague soft hyperrings.

In this paper, we develop the initial theory of vague soft hyperrings in Rosenfeld's sense as a continuation to the theory of vague soft hypergroups introduced in [10]. Subsequently some of the fundamental properties and structural characteristics of these concepts are studied and discussed. Lastly, we prove that there exists a one-to-one correspondence between some of these concepts and their corresponding concepts in soft hyperring theory as well as classical hyperring theory.

## 2. Preliminaries

In this section, some well-known and useful definitions pertaining to the development of the theory of vague soft hyperrings introduced here will be presented. These definitions and concepts will be used throughout this chapter.

**Definition 1 (see [6]).**

A pair (*F*, *A*) is called a* soft set* over *U*, where *F* is a mapping given by *F* : *A* → *P*(*U*). In other words, a soft set over *U* is a parameterized family of subsets of the universe *U*. For *ε* ∈ *A*, *F*(*ε*) may be considered as the set of *ε*-elements of the soft set (*F*, *A*) or as the *ε*-approximate elements of the soft set.

**Definition 2 (see [6]).**

For a soft set (*F*, *A*), the set Supp(*F*, *A*) = {*x* ∈ *A* : *F*(*x*) ≠ *∅*} is called the* support* of the soft set (*F*, *A*). Thus a* null soft set* is a soft set with an empty support and a soft set (*F*, *A*) is said to be nonnull if Supp(*F*, *A*) ≠ *∅*.

**Definition 3 (see [11]).**

Let *X* be a space of points (objects) with a generic element of *X* denoted by *x*. A* vague set V* in *X* is characterized by a truth membership function *t*_*V*_ : *X* → [0, 1] and a false membership function *f*_*V*_ : *X* → [0, 1]. The value *t*_*V*_(*x*) is a lower bound on the grade of membership of *x* derived from the evidence for *x* and *f*_*V*_(*x*) is a lower bound on the negation of *x* derived from the evidence against *x*. The values *t*_*V*_(*x*) and *f*_*V*_(*x*) both associate a real number in the interval [0, 1] with each point in *X*, where *t*_*V*_(*x*) + *f*_*V*_(*x*) ≤ 1. This approach bounds the grade of membership of *x* to a subinterval [*t*_*V*_(*x*), 1 − *f*_*V*_(*x*)] of [0, 1]. Hence a vague set is a form of fuzzy set, albeit a more accurate form of fuzzy set.

**Definition 4 (see [12]).**

A pair $\left(\hat{F},A\right)$ is called a* vague soft set* over *U* where $\hat{F}$ is a mapping given by $\hat{F}:A\to V(U)$ and *V*(*U*) is the power set of vague sets over *U*. In other words, a vague soft set over *U* is a parameterized family of vague sets of the universe *U*. Every set $\hat{F}(e)$, for all *e* ∈ *A*, from this family may be considered as the set of *e*-approximate elements of the vague soft set $\left(\hat{F},A\right)$. Hence the vague soft set $\left(\hat{F},A\right)$ can be viewed as consisting of a collection of approximations of the following form:

(1) F^,A=F^axi:  i=1,2,3,…=tF^eixi,1−fF^eixixi:  i=1,2,3,…,

where ${\hat{F}}_{a}\left(x_{i}\right)$ is a subset of $\left(\hat{F},A\right)$ for all *e* ∈ *A* and for all *x* ∈ *U*.

**Example 5 (see [12]).**

Consider a vague soft set $\left(\hat{F},E\right)$, where *U* is a set of six houses under consideration of a decision maker to purchase which is denoted by *U* = {*h*_1_, *h*_2_, *h*_3_, *h*_4_, *h*_5_, *h*_6_} and *E* is a parameter set, where *E* = {*e*_1_, *e*_2_, *e*_3_, *e*_4_, *e*_5_} = {expensive, beautiful, wooden, cheap, in  the  green  surroundings}. The vague soft set $\left(\hat{F},E\right)$ describes the “attractiveness of the houses” to this decision maker.

Suppose that

(2) F^e1=0.1,0.2h1,0.9,1h2,0.3,0.5h3,0.8,0.9h4,0.2,0.4h5,0.4,0.6h6,F^e2=0.9,1h1,0.2,0.7h2,0.6,0.9h3,0.2,0.4h4,0.3,0.4h5,0.1,0.6h6,F^e3=0,0h1,0,0h2,1,1h3,1,1h4,1,1h5,0,0h6,F^e4=0.8,0.9h1,0,0.1h2,0.5,0.7h3,0.1,0.2h4,0.6,0.8h5,0.4,0.6h6,F^e5=0.9,1h1,0.2,0.3h2,0.1,0.4h3,0.1,0.2h4,0.2,0.4h5,0.7,0.9h6.

The vague soft set $\left(\hat{F},E\right)$ is a parameterized family $\left\{\hat{F}\left(e_{i}\right),\;\;i=1,2,3,4,5\right\}$ of vague sets on *U*, and

(3) F^,E=Expensive  Houses=0.1,0.2h1,0.9,1h2,0.3,0.5h3,0.8,0.9h4,0.2,0.4h5,0.4,0.6h6,  Beautiful  Houses=0.9,1h1,0.2,0.7h2,0.6,0.9h3,0.2,0.4h4,0.3,0.4h5,0.1,0.6h6,….

**Definition 6 (see [10]).**

Let $\left(\hat{F},A\right)$ be a vague soft set over *X*. The* support* of $\left(\hat{F},A\right)$ denoted by $Supp(\hat{F},A)$ is defined as

(4) Supp⁡F^,A=a∈A:F^ax≠∅^,  i.e.,  tF^ax≠0,  1−fF^ax≠0,

for all *x* ∈ *X*.

It is to be noted that a* null vague soft set* is a vague soft set where both the truth and false membership functions are equal to zero. Therefore, a vague soft set $\left(\hat{F},A\right)$ is said to be nonnull if $Supp(\hat{F},A)\neq \emptyset$.

**Definition 7 (see [12]).**

Let $\left(\hat{F},A\right)$ and $\left(\hat{G},B\right)$ be vague soft sets over *U*. The set $\left(\hat{F},A\right)$ is called a* vague soft subset* of $\left(\hat{G},B\right)$ if *A*⊆*B* and, for every *a* ∈ *A*, $\hat{F}(a)$ and $\hat{G}(a)$ are the same approximation. In this case, $\left(\hat{G},B\right)$ is called the* vague soft superset* of $\left(\hat{F},A\right)$ and this relationship is denoted by $\left(\hat{F},A\right)\subseteq \left(\hat{G},B\right)$.

**Definition 8 (see [12]).**

If $\left(\hat{F},A\right)$ and $\left(\hat{G},B\right)$ are two vague soft sets over *U*, the* intersection* of $\left(\hat{F},A\right)$ and $\left(\hat{G},B\right)$ denoted as “$\left(\hat{F},A\right)\;\tilde{\cap }\;\left(\hat{G},B\right)$” is defined by $\left(\hat{F},A\right)\;\tilde{\cap }\;\left(\hat{G},B\right)=\left(\hat{H},A\times B\right)$, where

(5) tH^cx=tF^cx,c∈A−B,tG^cx,c∈B−A,min⁡tF^cx,tG^cx,c∈B∩A,1−fH^cx=1−fF^cx,c∈A−B,1−fG^cx,c∈B−A,min⁡1−fF^cx,1−fG^cx,c∈B∩A,

for all $c\in Supp\left(\hat{H},C\right)$ and for all *x* ∈ *U*.

**Definition 9 (see [12]).**

Let $\left(\hat{F},A\right)$ and $\left(\hat{G},B\right)$ be vague soft sets over *U*. Then “$\left(\hat{F},A\right)\;AND\;\left(\hat{G},B\right)$” denoted by $\left(\hat{F},A\right)\;\tilde{\wedge }\;\left(\hat{G},B\right)$ is defined as $\left(\hat{F},A\right)\;\tilde{\wedge }\;\left(\hat{G},B\right)=\left(\hat{H},A\times B\right)$, where

(6) tH^α,βx=min⁡tF^αx,tG^βx,1−fH^α,βx=min⁡1−fF^αx,1−fG^βx,

for every (*α*, *β*) ∈ *A* × *B* and *x* ∈ *U*, where $t_{{\hat{H}}_{\left(\alpha ,\beta \right)}}$ and $f_{{\hat{H}}_{\left(\alpha ,\beta \right)}}$ are the truth membership function and false membership function of ${\hat{H}}_{\left(\alpha ,\beta \right)}$, respectively. This relationship can be written as ${\hat{H}}_{\left(\alpha ,\beta \right)}\left(x\right)={\hat{F}}_{\alpha }\left(x\right)\cap {\hat{G}}_{\beta }\left(x\right)$, where ∩ represents the intersection operation between two vague soft sets as defined in the case of *c* ∈ *B*∩*A* in Definition 8.

**Definition 10 (see [10]).**

Let $\left(\hat{F},A\right)$ be a vague soft set over *U*. Then, for all *α*, *β* ∈ [0, 1], where *α* ≤ *β*, the (*α*, *β*)*-cut* or the* vague soft *(*α*, *β*)*-cut* of $\left(\hat{F},A\right)$ is a subset of *U* which is as defined below:

(7) F^,Aα,β=x∈U:tF^ax≥α,  1−fF^ax≥β,  i.e.,  F^ax≥α,β,

for all *a* ∈ *A*.

If *α* = *β*, then it is called the* vague soft*  (*α*, *α*)*-cut* of $\left(\hat{F},A\right)$ or the *α-level set* of $\left(\hat{F},A\right)$, denoted by ${\left(\hat{F},A\right)}_{\left(\alpha ,\alpha \right)}$, is a subset of *U* which is as defined below:

(8) F^,Aα,α=x∈U:tF^ax≥α,  1−fF^ax≥α,  i.e.,  F^ax≥α,α,

for all *a* ∈ *A*.

**Definition 11 (see [10]).**

Let $\left(\hat{F},A\right)$ be a vague soft set over *X* and let *G* be a nonnull subset of *X*. Then ${\left(\hat{F},A\right)}_{G}$ is called a* vague soft characteristic set* over *G* in [0, 1] and the lower bound and upper bound of ${\left({\hat{F}}_{a}\right)}_{G}$ are as defined below:

(9) $$\begin{array}{c} t_{{\left({\hat{F}}_{a}\right)}_{G}}\left(\;x\;\right)=1-f_{{\left({\hat{F}}_{a}\right)}_{G}}\left(\;x\;\right)=\left\{\begin{array}{cc} s, & if\;x\in G,\\ w, & otherwise, \end{array}\right. \end{array}$$

where ${\left({\hat{F}}_{a}\right)}_{G}$ is a subset of ${\left(\hat{F},A\right)}_{G}$, *x* ∈ *X*, *s*, *w* ∈ [0, 1], and *s* > *w*.

This paper pertains to the application of classical hyperring theory to the concept of vague soft sets. Furthermore, the relationships that exist between the concepts introduced in this paper and the corresponding concepts in classical hyperring theory are also studied and discussed. The definitions of several important concepts pertaining to the theory of hyperstructures are presented below.

**Definition 12 (see [1]).**

A* hypergroup *〈*H*, ∘〉 is a set *H* equipped with an associative hyperoperation (∘) : *H* × *H* → *P*(*H*) which satisfies the reproduction axiom given by *x*∘*H* = *H*∘*x* = *H* for all *x* in *H*.

**Definition 13 (see [13]).**

A hyperstructure 〈*H*, ∘〉 is called an *H*_*v*_ *-group* if the following axioms hold:

- *x*∘(*y*∘*z*)∩(*x*∘*y*)∘*z* ≠ *∅* for all *x*, *y*, *z* ∈ *H*,
- *x*∘*H* = *H*∘*x* = *H* for all *x* in *H*.

If 〈*H*, ∘〉 only satisfies the first axiom, then it is called a *H*_*v*_ *-semigroup*.

**Definition 14 (see [1]).**

A subset *K* of *H* is called a* subhypergroup* if 〈*K*, ∘〉 is a hypergroup.

**Definition 15 (see [3]).**

A *H*_*ν*_-*ring* is a multivalued system (*R*, +, ∘) which satisfies the following axioms:

(i) (*R*, +) is a *H*_*ν*_-group,
(ii) (*R*, ∘) is a *H*_*ν*_-semigroup,
(iii) the hyperoperation “∘” is weak distributive over the hyperoperation “+”; that is, for each *x*, *y*, *z* ∈ *R* the following holds true:
  (10) x∘y+z∩x∘y+x∘z≠ϕ,x+y∘z∩x∘z+y∘z≠ϕ.

**Definition 16 (see [3]).**

A nonempty subset *R*′ of *R* is called a* subhyperring* of (*R*, + , ∘) if (*R*′, +) is a subhypergroup of (*R*, +) and for all *x*, *y*, *z* ∈ *R*′, *x*∘*y* ∈ *P* ^*∗*^(*R*′), where *P* ^*∗*^(*R*′) is the set of all nonempty subsets of *R*′.

**Definition 17 (see [3]).**

Let *R* be a *H*_*v*_-ring. A nonempty subset *I* of *R* is called a* left *(resp.,* right*) *H*_*v*_ *-ideal* if the following axioms hold:

- (*I*, +) is a *H*_*v*_-subgroup of (*R*, +),
- *R*∘*I*⊆*I* (resp.  *I*∘*R*⊆*I*).

If *I* is both left and right *H*_*v*_-ideals of *R*, then *I* is said to be a *H*_*v*_ *-ideal* of *R*.

## 3. Vague Soft Hyperrings

In this section, the concept of vague soft hyperrings in Rosenfeld's sense is introduced as a continuation to the theory of vague soft hypergroups introduced by [10]. We derive the definition of vague soft hyperrings by applying the theory of hyperrings to vague soft sets. The properties of this concept are studied and discussed.

From now on, let (*R*, + , ∘) be a hyperring (or *H*_*ν*_-ring), let *E* be a set of parameters, and let *A*⊆*E*. For the sake of simplicity, we will denote (*R*, + , ∘) by *R*.

**Definition 18 (see [14]).**

Let (*F*, *A*) be a nonnull soft set over *R*. Then (*F*, *A*) is called a* soft hyperring* over *R* if *F*(*x*) is a subhyperring of *R* for all $x\in Supp\left(\hat{F},A\right)$.

**Definition 19 (see [10]).**

Let $\left(\hat{F},A\right)$ be a nonnull vague soft set over a hypergroup 〈*H*, ∘〉. Then $\left(\hat{F},A\right)$ is called a* vague soft semihypergroup* over 〈*H*, ∘〉 if the following conditions are satisfied for all $a\in Supp\left(\hat{F},A\right)$:

For all *x*, *y* ∈ 〈*H*, ∘〉, $\min\left\{t_{{\hat{F}}_{a}}\left(x\right),t_{{\hat{F}}_{a}}\left(y\right)\right\}\leq \inf\left\{t_{{\hat{F}}_{a}}\left(z\right):z\in x\circ y\right\}$ and $\min\left\{{1-f}_{{\hat{F}}_{a}}\left(x\right),{1-f}_{{\hat{F}}_{a}}\left(y\right)\right\}\leq \inf\left\{{1-f}_{{\hat{F}}_{a}}\left(z\right):z\in x\circ y\right\}$; that is, $\min\left\{{\hat{F}}_{a}\left(x\right),{\hat{F}}_{a}\left(y\right)\right\}\leq \inf\left\{{\hat{F}}_{a}\left(z\right):z\in x\circ y\right\}$.

**Definition 20 (see [10]).**

Let $\left(\hat{F},A\right)$ be a nonnull vague soft set over *H*. Then $\left(\hat{F},A\right)$ is called a* vague soft hypergroup* over *H* if the following conditions are satisfied for all $a\in Supp\left(\hat{F},A\right)$:

- For all $x,y\in H,\min\left\{t_{{\hat{F}}_{a}}\left(x\right),t_{{\hat{F}}_{a}}\left(y\right)\right\}\;\leq \inf\left\{t_{{\hat{F}}_{a}}\left(z\right):z\;\in \;x\circ y\right\}$ and $\min\{{1-f}_{{\hat{F}}_{a}}\left(x\right),{1-f}_{{\hat{F}}_{a}}(y)\}\;\leq inf\{1-f_{{\hat{F}}_{a}}\left(z\right):z\in x\circ y\}$; that is, $\min\left\{{\hat{F}}_{a}\left(x\right),{\hat{F}}_{a}\left(y\right)\right\}\leq \inf\left\{{\hat{F}}_{a}\left(z\right):z\in x\circ y\right\}$.
- For all *w*, *x* ∈ *H*, there exists *y* ∈ *H* such that *x* ∈ *w*∘*y* and min ⁡tF^aw,tF^ax≤tF^a(y) and min ⁡1-fF^aw,1-fF^ax≤1-fF^a(y); that is, min ⁡F^aw,F^ax≤F^ay.
- For all *w*, *x* ∈ *H*, there exists *z* ∈ *H* such that *x* ∈ *z*∘*w* and min ⁡tF^aw,tF^ax≤tF^a(z) and min ⁡1-fF^aw,1-fF^ax≤1-fF^a(z); that is, $\min\left\{{\hat{F}}_{a}\left(w\right),{\hat{F}}_{a}\left(x\right)\right\}\leq {\hat{F}}_{a}\left(z\right)$.

Conditions (ii) and (iii) represent the left and right reproduction axioms, respectively. Then ${\hat{F}}_{a}$ is a nonnull* vague subhypergroup* of 〈*H*, ∘〉 (in Rosenfeld's sense) for all $a\in Supp\left(\hat{F},A\right)$.

**Definition 21 .**

Let $\left(\hat{F},A\right)$ be a nonnull vague soft set over *R*. Then $\left(\hat{F},A\right)$ is called a* vague soft hyperring* over *R* if, for all $a\in Supp\left(\hat{F},A\right)$, the following conditions are satisfied:

- For all *x*, *y* ∈ *R*, $\min\left\{t_{{\hat{F}}_{a}}\left(x\right),t_{{\hat{F}}_{a}}\left(y\right)\right\}\leq \inf\left\{t_{{\hat{F}}_{a}}\left(z\right):z\in x+y\right\}$ and $\min\left\{{1-f}_{{\hat{F}}_{a}}\left(x\right),{1-f}_{{\hat{F}}_{a}}\left(y\right)\right\}\leq \inf\left\{{1-f}_{{\hat{F}}_{a}}\left(z\right):z\in x+y\right\}$, which means $\min\left\{{\hat{F}}_{a}\left(x\right),{\hat{F}}_{a}\left(y\right)\right\}\leq \inf\left\{{\hat{F}}_{a}\left(z\right):z\in x+y\right\}$.
- For all *w*, *x* ∈ *R*, there exists *y* ∈ *R* such that *x* ∈ *w* + *y* and $\min\left\{t_{{\hat{F}}_{a}}\left(w\right),t_{{\hat{F}}_{a}}\left(x\right)\right\}\leq t_{{\hat{F}}_{a}}(y)$ and $\min\left\{1-f_{{\hat{F}}_{a}}\left(w\right),{1-f}_{{\hat{F}}_{a}}\left(x\right)\right\}\leq 1-f_{{\hat{F}}_{a}}\left(y\right)$; that is, $\min\left\{{\hat{F}}_{a}\left(w\right),{\hat{F}}_{a}\left(x\right)\right\}\leq {\hat{F}}_{a}\left(y\right)$.
- For all *w*, *x* ∈ *R*, there exists *z* ∈ *R* such that *x* ∈ *z* + *w* and $\min\left\{t_{{\hat{F}}_{a}}\left(w\right),t_{{\hat{F}}_{a}}\left(x\right)\right\}\leq t_{{\hat{F}}_{a}}(z)$ and $\min\left\{{1-f}_{{\hat{F}}_{a}}\left(w\right),{1-f}_{{\hat{F}}_{a}}\left(x\right)\right\}\leq {1-f}_{{\hat{F}}_{a}}\left(z\right)$; that is, $\min\left\{{\hat{F}}_{a}\left(w\right),{\hat{F}}_{a}\left(x\right)\right\}\leq {\hat{F}}_{a}\left(z\right)$.
- For all *x*, *y* ∈ *R*, $\min\left\{t_{{\hat{F}}_{a}}\left(x\right),t_{{\hat{F}}_{a}}\left(y\right)\right\}\leq \inf\left\{t_{{\hat{F}}_{a}}\left(z\right):z\in x\circ y\right\}$ and $\min\left\{{1-f}_{{\hat{F}}_{a}}\left(x\right),{1-f}_{{\hat{F}}_{a}}\left(y\right)\right\}\leq \inf\left\{{1-f}_{{\hat{F}}_{a}}\left(z\right):z\in x\circ y\right\}$; that is, $\min\left\{{\hat{F}}_{a}\left(x\right),{\hat{F}}_{a}\left(y\right)\right\}\leq \inf\left\{{\hat{F}}_{a}\left(z\right):z\in x\circ y\right\}$.

That is, ${\hat{F}}_{a}$ is a nonnull* vague subhyperring* (in Rosenfeld's sense) of *R* for each $a\in Supp\left(\hat{F},A\right)$.

Conditions (i), (ii), and (iii) indicate that ${\hat{F}}_{a}$ is a vague subhypergroup of (*R*, +) while condition (iv) indicates that ${\hat{F}}_{a}$ is a vague subsemihypergroup of (*R*, ∘). Therefore it can be concluded that $\left(\hat{F},A\right)$ is a* vague soft hyperring* over *R* if $\left(\hat{F},A\right)$ is a vague soft hypergroup over (*R*, +) and a vague soft semihypergroup over (*R*, ∘).

**Theorem 22 .**

Let $\left(\hat{F},A\right)$ be a nonnull vague soft set over *R*. Then the necessary and sufficient condition for $\left(\hat{F},A\right)$ to be a vague soft hyperring over *R* is for $\left(\hat{F},A\right)$ to be a vague soft semihypergroup over (*R*, ∘) and also a vague soft hypergroup over (*R*, +).

**Proof**

The proof is straightforward by Definition 21.

**Example 23 .**

A vague soft hyperring $\left(\hat{F},A\right)$ for which *A* is a singleton is a vague subhyperring. Hence vague subhyperrings and classical hyperrings are a particular type of vague soft hyperrings.

**Example 24 .**

Let $\left(\hat{F},A\right)$ be a nonnull vague soft set over a hyperring *R* which is as defined below:

(11) F^,A+=F^a+=x∈R:tF^a+x=tF^ax+1−tF^ae,1−fF^a+x=1−fF^ax+fF^ae,  i.e.,  F^a+x=F^ax+1−F^ae.

Then ${\left(\hat{F},A\right)}^{+}$ is a vague soft hyperring over *R*.

**Example 25 .**

Let ${\hat{F}}_{a}$ be a vague subhyperring of a hyperring *R*. This means that ${\hat{F}}_{a}$ satisfies the axioms stated in Definition 21. Now consider the family of *α*-level sets for ${\hat{F}}_{a}$ given by

(12) $$\begin{array}{c} {\left(\;\hat{F},A\;\right)}_{\alpha }=\left\{\;x\in H:{\hat{F}}_{a}\left(\;x\;\right)\geq \alpha \;\right\}, \end{array}$$

for all *a* ∈ *A* and *α* ∈ [0, 1]. Then, for all *α* ∈ [0, 1], ${\hat{F}}_{a}$ is a subhyperring of *R*. Hence $\left(\hat{F},\left[0,1\right]\right)$ is a soft hyperring over *R*.

**Example 26 .**

Any soft hyperring is a vague subhyperring since any characteristic function of a subhyperring is a vague subhyperring.

**Proposition 27 .**

Let $\left(\hat{F},A\right)$ and $\left(\hat{G},B\right)$ be vague soft hyperrings over *R*. Then $\left(\hat{F},A\right)\;\tilde{\wedge }\;(\hat{G},B)$ is a vague soft hyperring over *R* if it is nonnull.

**Proof**

Suppose that $\left(\hat{F},A\right)$ and $\left(\hat{G},B\right)$ are vague soft hyperrings over *R*. Now let $\left(\hat{F},A\right)\;\tilde{\wedge }\;\left(\hat{G},B\right)=\left(\hat{H},A\times B\right)$. Then for all $(\alpha ,\beta )\in Supp\left(\hat{H},A\times B\right)$ and for all *x* ∈ *R* we have

(13) tH^α,βx=min⁡tF^αx,tG^βx,1−fH^α,βx=min⁡1−fF^αx,1−fG^βx;

that is,

(14) $$\begin{array}{c} {\hat{H}}_{\left(\alpha ,\beta \right)}\left(\;x\;\right)={\hat{F}}_{\alpha }\left(\;x\;\right)\cap {\hat{G}}_{\beta }\left(\;x\;\right). \end{array}$$

Since $\left(\hat{F},A\right)$ and $\left(\hat{G},B\right)$ are both nonnull and $\left(\hat{H},A\times B\right)$ represents the basic intersection between $\left(\hat{F},A\right)$ and $\left(\hat{G},B\right)$ it follows that $\left(\hat{H},A\times B\right)$ must be nonnull. For the sake of similarity, we only show the proof for the truth membership function of ${\hat{H}}_{\left(\alpha ,\beta \right)}$ given by $t_{{\hat{H}}_{\left(\alpha ,\beta \right)}}$. The proof for $1-f_{{\hat{H}}_{\left(\alpha ,\beta \right)}}$ can be derived in the same manner.

Hence for all *x*, *y* ∈ *R* and $(\alpha ,\beta )\in Supp\left(\hat{H},A\times B\right)$ we obtain

(15) min⁡tH^α,βx,tH^α,βy=min⁡tF^αx∩tG^βx,tF^αy∩tG^βy≤min⁡tF^αx∩tF^αy,tG^βx∩tG^βy≤min⁡tF^αx,tF^αy∩min⁡tG^βx,tG^βy≤inf⁡tF^αz:z∈x+y∩inf⁡tG^βz:z∈x+y≤inf⁡tF^αz∩tG^βz:z∈x+y=inf⁡tH^α,β:z∈x+y.

Furthermore for all *w*, *x* ∈ *R* we have

(16) min⁡tH^α,βw,tH^α,βx=min⁡tF^αw∩tG^βw,tF^αx∩tG^βx≤min⁡tF^αw∩tF^αx,tG^βw∩tG^βx≤min⁡tF^αw,tF^αx∩min⁡tG^βw,tG^βx≤tF^αy∩tG^βy=tH^α,βy,

where *y* ∈ *R* such that *x* ∈ *w* + *y*. Similarly, it can also be proved that $\min\left\{t_{{\hat{H}}_{\left(\alpha ,\beta \right)}}\left(w\right),t_{{\hat{H}}_{\left(\alpha ,\beta \right)}}\left(x\right)\right\}\leq t_{{\hat{H}}_{\left(\alpha ,\beta \right)}}\left(z\right)$, where *z* ∈ *R* such that *x* ∈ *z* + *w*. Therefore it has been proved that ${\hat{H}}_{\left(\alpha ,\beta \right)}$ is a vague subhypergroup of (*R*, +), which means that $\left(\hat{H},A\times B\right)$ is a vague soft hypergroup over (*R*, +).

Lastly for all *x*, *y* ∈ *R* we have

(17) min⁡tH^α,βx,tH^α,βy=min⁡tF^αx∩tG^βx,tF^αy∩tG^βy≤min⁡tF^αx∩tF^αy,tG^βx∩tG^βy≤min⁡tF^αx,tF^αy∩min⁡tG^βx,tG^βy≤inf⁡tF^αz:z∈x∘y∩inf⁡tG^βz:z∈x∘y≤inf⁡tF^αz∩tG^βz:z∈x∘y=inf⁡tH^α,β:z∈x∘y.

This proves that ${\hat{H}}_{\left(\alpha ,\beta \right)}$ is a vague subhyperring of *R* for all $\left(\alpha ,\beta \right)\in Supp\left(\hat{H},A\times B\right)$. Hence $\left(\hat{H},A\times B\right)=\left(\hat{F},A\right)\;\tilde{\wedge }\;(\hat{G},B)$ is a vague soft hyperring over *R*.

**Theorem 28 .**

Let $\left(\hat{F},A\right)$ be a vague soft set over *R*. Then $\left(\hat{F},A\right)$ is a vague soft hyperring over *R* if and only if, for every *t* ∈ [0, 1], ${\left(\hat{F},A\right)}_{\left(t,t\right)}$ is a soft hyperring over *R*.

**Proof**

(⇒) Since *R* is a hyperring, (*R*, +) is a commutative hypergroup and (*R*, ∘) is a semihypergroup. Let $\left(\hat{F},A\right)$ be a vague soft hyperring over (*R*, + , ∘) and *t* ∈ [0, 1]. Then, for all $a\in Supp\left(\hat{F},A\right)$, the corresponding ${\hat{F}}_{a}$ is a vague subhyperring of *R*. Now let $x,y\in {\left({\hat{F}}_{a}\right)}_{\left(t,t\right)}.$ Then $t_{{\hat{F}}_{a}}\left(x\right),t_{{\hat{F}}_{a}}(y)\geq t$ and ${1-f}_{{\hat{F}}_{a}}\left(x\right)$, ${1-f}_{{\hat{F}}_{a}}\left(y\right)\geq t$, which means that ${\hat{F}}_{a}\left(x\right)\geq t$ and ${\hat{F}}_{a}\left(y\right)\geq t$. Furthermore since ${\hat{F}}_{a}$ is a vague subhypergroup of (*R*, +), we have $\inf\left\{{\hat{F}}_{a}\left(z\right):z\in x+y\right\}\geq \min\left\{{\hat{F}}_{a}\left(x\right),{\hat{F}}_{a}\left(y\right)\right\}\geq t$. This implies that $z\in {\left({\hat{F}}_{a}\right)}_{\left(t,t\right)}$ and therefore, for every *z* ∈ *x* + *y*, we obtain $x+y\subseteq {\left({\hat{F}}_{a}\right)}_{\left(t,t\right)}$. As such, for every $z\in {\left({\hat{F}}_{a}\right)}_{\left(t,t\right)}$, we obtain $z+{\left({\hat{F}}_{a}\right)}_{\left(t,t\right)}\subseteq {\left({\hat{F}}_{a}\right)}_{\left(t,t\right)}$. Now let $x,z\in {\left({\hat{F}}_{a}\right)}_{\left(t,t\right)}$. Then $t_{{\hat{F}}_{a}}\left(x\right),t_{{\hat{F}}_{a}}(z)\geq t$ and ${1-f}_{{\hat{F}}_{a}}\left(x\right),{1-f}_{{\hat{F}}_{a}}\left(z\right)\geq t$, which means that ${\hat{F}}_{a}\left(x\right)\geq t$ and ${\hat{F}}_{a}\left(z\right)\geq t$. Due to the fact that ${\hat{F}}_{a}$ is a vague subhypergroup of (*R*, +), there exists *y* ∈ *R* such that *x* ∈ *z* + *y* and $\min\left\{{\hat{F}}_{a}\left(z\right),{\hat{F}}_{a}\left(x\right)\right\}\leq {\hat{F}}_{a}\left(y\right)$. Since $x,z\in {\left({\hat{F}}_{a}\right)}_{\left(t,t\right)}$, we obtain $\min\left\{{\hat{F}}_{a}\left(z\right),{\hat{F}}_{a}\left(x\right)\right\}\geq \min\left\{t,t\right\}=t$ and this implies that ${\hat{F}}_{a}(y)\geq t$ and as a result, $y\in {\left({\hat{F}}_{a}\right)}_{\left(t,t\right)}.$ Therefore, we obtain ${\left({\hat{F}}_{a}\right)}_{\left(t,t\right)}\subseteq z+{\left({\hat{F}}_{a}\right)}_{\left(t,t\right)}$. As such, we obtain $z+{\left({\hat{F}}_{a}\right)}_{\left(t,t\right)}={\left({\hat{F}}_{a}\right)}_{\left(t,t\right)}$. As a result, ${\left({\hat{F}}_{a}\right)}_{\left(t,t\right)}$ is a subhypergroup of (*R*, +). Hence ${\left(\hat{F},A\right)}_{\left(t,t\right)}$ is a soft hypergroup over (*R*, +). Furthermore, since (*R*, ∘) is a semihypergroup, ${\hat{F}}_{a}$ is a nonnull vague subsemihypergroup of (*R*, ∘) for all $a\in Supp\left(\hat{F},A\right)$ and $\min\left\{{\hat{F}}_{a}\left(x\right),{\hat{F}}_{a}\left(y\right)\right\}\leq \inf\left\{{\hat{F}}_{a}\left(z\right):z\in x\circ y\right\}$. Now let $x,y\in {\left({\hat{F}}_{a}\right)}_{\left(t,t\right)}$. Then $t_{{\hat{F}}_{a}}\left(x\right),t_{{\hat{F}}_{a}}(y)\geq t$ and ${1-f}_{{\hat{F}}_{a}}\left(x\right),{1-f}_{{\hat{F}}_{a}}\left(y\right)\geq t$, which means that ${\hat{F}}_{a}\left(x\right)\geq t$ and ${\hat{F}}_{a}\left(y\right)\geq t$. Therefore it follows that $\min\left\{{\hat{F}}_{a}\left(x\right),{\hat{F}}_{a}\left(y\right)\right\}\geq \min\left\{t,t\right\}=t$ and this implies that $\inf\left\{{\hat{F}}_{a}\left(z\right):z\in x\circ y\right\}\geq t$. This in turn implies that $z\in {\left({\hat{F}}_{a}\right)}_{\left(t,t\right)}$ and consequently $x\circ y\subseteq {\left({\hat{F}}_{a}\right)}_{\left(t,t\right)}$. Therefore, for every $x,y\in {\left({\hat{F}}_{a}\right)}_{\left(t,t\right)}$, we obtain *x*∘*y* ∈ *P* ^*∗*^(*R*). As such, ${\left({\hat{F}}_{a}\right)}_{\left(t,t\right)}$ is a subhyperring of *R*. Hence ${\left(\hat{F},A\right)}_{\left(t,t\right)}$ is a soft hyperring over *R*.

(⇐) Assume that ${\left(\hat{F},A\right)}_{\left(t,t\right)}$ is a soft hyperring over *R*. Thus for every *t* ∈ [0, 1], ${\left({\hat{F}}_{a}\right)}_{\left(t,t\right)}$ is a nonnull subhyperring of *R*. Now for every *x*, *y* ∈ *R*, we have $\min\left\{{\hat{F}}_{a}\left(x\right),{\hat{F}}_{a}\left(y\right)\right\}\leq {\hat{F}}_{a}(x)$ or $\min\left\{{\hat{F}}_{a}\left(x\right),{\hat{F}}_{a}\left(y\right)\right\}\leq {\hat{F}}_{a}\left(y\right)$. So if we let $\min\left\{{\hat{F}}_{a}\left(x\right),{\hat{F}}_{a}\left(y\right)\right\}=t_{0}$, then $x,y\in {\left({\hat{F}}_{a}\right)}_{\left(t_{0},t_{0}\right)}$ and therefore $x+y\subseteq {\left({\hat{F}}_{a}\right)}_{\left(t_{0},t_{0}\right)}$. Therefore, for every *z* ∈ *x* + *y*, we have ${\hat{F}}_{a}(z)\geq t_{0}$ which implies that $\min\left\{{\hat{F}}_{a}\left(x\right),{\hat{F}}_{a}\left(y\right)\right\}\leq \inf\left\{{\hat{F}}_{a}\left(z\right):z\in x+y\right\}$. As such, condition (i) of Definition 21 is verified. Next, let *w*, *x* ∈ *R* and $\min\left\{{\hat{F}}_{a}\left(w\right),{\hat{F}}_{a}\left(x\right)\right\}=t_{1}$. This implies that $w,x\in {\left({\hat{F}}_{a}\right)}_{\left(t_{1},t_{1}\right)}$ which means that there exists $y\in {\left({\hat{F}}_{a}\right)}_{\left(t_{1},t_{1}\right)}$ such that *x* ∈ *w*∘*y*. Since $y\;\in {\left({\hat{F}}_{a}\right)}_{\left(t_{1},t_{1}\right)}$, we have $t_{{\hat{F}}_{a}}(y)\geq t_{1}$ and $1-t_{{\hat{F}}_{a}}(y)\geq t_{1}$ which means that ${\hat{F}}_{a}(y)\geq t_{1}$ and thus $\min\left\{{\hat{F}}_{a}\left(w\right),{\hat{F}}_{a}\left(x\right)\right\}=t_{1}\leq {\hat{F}}_{a}(y)$ which means that $\min\left\{{\hat{F}}_{a}\left(w\right),{\hat{F}}_{a}\left(x\right)\right\}\leq {\hat{F}}_{a}\left(y\right)$. Therefore condition (ii) of Definition 21 has been verified. Condition (iii) of Definition 21 can be verified in a similar manner. Thus it has been proven that ${\hat{F}}_{a}$ is a vague subhypergroup of (*R*, +). Now ${\left({\hat{F}}_{a}\right)}_{\left(t,t\right)}$ is a subsemihypergroup of the semihypergroup (*R*, ∘). Let $\min\left\{{\hat{F}}_{a}\left(x\right),{\hat{F}}_{a}\left(y\right)\right\}=t_{2}$ for every *x*, *y* ∈ *R*. Thus $x,y\in {\left({\hat{F}}_{a}\right)}_{\left(t_{2},t_{2}\right)}$ and therefore we have $x\circ y\;\subseteq \;{\left({\hat{F}}_{a}\right)}_{\left(t_{2},t_{2}\right)}$. Thus, for every *z* ∈ *x*∘*y*, we obtain ${\hat{F}}_{a}\left(z\right)\geq t_{2}$. As a result, we obtain $\min\left\{{\hat{F}}_{a}\left(x\right),{\hat{F}}_{a}\left(y\right)\right\}\leq \inf\left\{{\hat{F}}_{a}\left(z\right):z\in x\circ y\right\}$ which proves that condition (iv) of Definition 21 has been verified. As such, ${\hat{F}}_{a}$ is a vague subhyperring of *R* and therefore $\left(\hat{F},A\right)$ is a vague soft hyperring over *R*.

Theorem 28 proves that there exists a one-to-one correspondence between vague soft hyperrings and soft hyperrings over a hyperring.

**Theorem 29 .**

Let $\left(\hat{F},A\right)$ be a vague soft hyperring over *R*. Then, for each *α*, *β* ∈ [0, 1], ${\left(\hat{F},A\right)}_{\left(\alpha ,\beta \right)}$ is a subhyperring of *R*.

**Proof**

Let $\left(\hat{F},A\right)$ be a vague soft hyperring over (*R*, +, ∘). Then for each $a\in Supp\left(\hat{F},A\right)$, the corresponding ${\hat{F}}_{a}$ is a vague subhyperring of *R*. Now let $x,y\in {\left(\hat{F},A\right)}_{\left(\alpha ,\beta \right)}$. Then $t_{{\hat{F}}_{a}}\left(x\right)\geq \alpha$, $1-f_{{\hat{F}}_{a}}(x)\geq \beta$ and $t_{{\hat{F}}_{a}}\left(y\right)\geq \alpha$, $1-f_{{\hat{F}}_{a}}\left(y\right)\geq \beta$. Thus the following is obtained:

(18) min⁡tF^ax,tF^ay≥min⁡α,α=α,min⁡1−fF^ax,1−fF^ay≥min⁡β,β=β.

However, since ${\hat{F}}_{a}$ is a vague subhypergroup of (*R*, +), we have $\inf\left\{t_{{\hat{F}}_{a}}\left(z\right):z\in x+y\right\}\geq \alpha$ and $\inf\left\{{1-f}_{{\hat{F}}_{a}}\left(z\right):z\in x+y\right\}\geq \beta$. Therefore, for every *z* ∈ *x* + *y*, we obtain $z\in {\left(\hat{F},A\right)}_{\left(\alpha ,\beta \right)}$ and thus $x+y\subseteq {\left(\hat{F},A\right)}_{\left(\alpha ,\beta \right)}$. As such, it can be concluded that $z+{\left(\hat{F},A\right)}_{\left(\alpha ,\beta \right)}\subseteq {\left(\hat{F},A\right)}_{\left(\alpha ,\beta \right)}$. Furthermore, since ${\hat{F}}_{a}$ is a vague subhypergroup of (*R*, +), there exists *y* ∈ *R* such that *x* ∈ *z* + *y* and $\min\left\{{\hat{F}}_{a}\left(x\right),{\hat{F}}_{a}\left(z\right)\right\}\leq {\hat{F}}_{a}\left(y\right)$. Now let $x,z\in {\left(\hat{F},A\right)}_{\left(\alpha ,\beta \right)}$. Then the following is obtained:

(19) min⁡tF^ax,tF^az≥min⁡α,α=α,min⁡1−fF^ax,1−fF^ay≥min⁡β,β=β.

This means that $t_{{\hat{F}}_{a}}(y)\geq \alpha$ and ${1-f}_{{\hat{F}}_{a}}(y)\geq \beta$ which implies that $y\in {\left(\hat{F},A\right)}_{\left(\alpha ,\beta \right)}$. Therefore for all $x,z\in {\left(\hat{F},A\right)}_{\left(\alpha ,\beta \right)}$, we have *x* ∈ *z* + *y* and $y\in {\left(\hat{F},A\right)}_{\left(\alpha ,\beta \right)}$. As such, it can be concluded that ${\left(\hat{F},A\right)}_{\left(\alpha ,\beta \right)}\subseteq z+{\left(\hat{F},A\right)}_{\left(\alpha ,\beta \right)}$ and this implies that, for any $z\in {\left(\hat{F},A\right)}_{\left(\alpha ,\beta \right)}$, $z+{\left(\hat{F},A\right)}_{\left(\alpha ,\beta \right)}={\left(\hat{F},A\right)}_{\left(\alpha ,\beta \right)}$. Hence it has been proven that ${\left(\hat{F},A\right)}_{\left(\alpha ,\beta \right)}$ is a subhypergroup of (*R*, +). Furthermore (*R*, ∘) is a semihypergroup and ${\hat{F}}_{a}$ is also a vague subsemihypergroup of (*R*, ∘) for all $a\in Supp\left(\hat{F},A\right)$. Now let $x,y\in {\left(\hat{F},A\right)}_{\left(\alpha ,\beta \right)}$. Then $\min\left\{t_{{\hat{F}}_{a}}\left(x\right),t_{{\hat{F}}_{a}}\left(y\right)\right\}\geq \min\left\{\alpha ,\alpha \right\}=\alpha$ and $\min\left\{{1-f}_{{\hat{F}}_{a}}\left(x\right),{1-f}_{{\hat{F}}_{a}}\left(y\right)\right\}\geq \min\left\{\beta ,\beta \right\}=\beta$ and this results in

(20) inf⁡tF^az:z∈x∘y≥min⁡tF^ax,tF^ay≥α,inf⁡1−fF^az:z∈x∘y≥min⁡1−fF^ax,1−fF^ay≥β.

This implies that, for all *z* ∈ *x*∘*y*, we obtain $z\in {\left(\hat{F},A\right)}_{\left(\alpha ,\beta \right)}$ and therefore $x\circ y\subseteq {\left(\hat{F},A\right)}_{\left(\alpha ,\beta \right)}$. Thus, for every $x,y\in {\left(\hat{F},A\right)}_{\left(\alpha ,\beta \right)}$, we obtain $x\circ y\in P^{*}\left({\left(\hat{F},A\right)}_{\left(\alpha ,\beta \right)}\right)$. As such, ${\left(\hat{F},A\right)}_{\left(\alpha ,\beta \right)}$ is a subsemihypergroup of the semihypergroup (*R*, ∘). Hence ${\left(\hat{F},A\right)}_{\left(\alpha ,\beta \right)}$ is a subhyperring of *R*.

As a result of Theorem 29, we obtain Corollary 30.

**Corollary 30 .**

Let $\left(\hat{F},A\right)$ be a vague soft hyperring over *R*. Then, for each *α* ∈ [0, 1], ${\left(\hat{F},A\right)}_{\left(\alpha ,\alpha \right)}$ is a subhyperring of *R*.

**Theorem 31 .**

Let *T* be a nonempty subset of *R*, let $\left(\hat{F},A\right)$ be a nonnull vague soft set over *R*, and let ${\left(\hat{F},A\right)}_{T}$ be a vague soft characteristic set over *T*. If $\left(\hat{F},A\right)$ is a vague soft hyperring over *R*, then *T* is a subhyperring of *R*.

**Proof**

Let $\left(\hat{F},A\right)$ be a vague soft hyperring over *R*. Then, by Definition 21, $\left(\hat{F},A\right)$ is a vague soft hypergroup over (*R*, +) and a vague soft semihypergroup over (*R*, ∘) and for each $a\in Supp\left(\hat{F},A\right)$, the corresponding vague subset ${\hat{F}}_{a}$ is a vague subhyperring of *R*. Furthermore, since (*R*, + , ∘) is a hyperring, (*R*, +) is a commutative hypergroup and (*R*, ∘) is a semihypergroup. Now let *x*, *y* ∈ *T*. Then $t_{{\left({\hat{F}}_{a}\right)}_{T}}\left(x\right)=t_{{\left({\hat{F}}_{a}\right)}_{T}}\left(y\right)=s$ and $f_{{\left({\hat{F}}_{a}\right)}_{T}}\left(x\right)=f_{{\left({\hat{F}}_{a}\right)}_{T}}\left(y\right)=w$, which means that ${\left({\hat{F}}_{a}\right)}_{T}\left(x\right)={\left({\hat{F}}_{a}\right)}_{T}\left(y\right)=s$. Therefore, $\min\left\{{\left({\hat{F}}_{a}\right)}_{T}\left(x\right),{\left({\hat{F}}_{a}\right)}_{T}\left(y\right)\right\}=\min\left\{s,s\right\}=s$ and so $\inf\left\{{\left({\hat{F}}_{a}\right)}_{T}\left(z\right):z\in x+y\right\}\geq s$ ($∵{\hat{F}}_{a}$ is a vague subhyperring of *R* and a vague subhypergroup of (*R*, +)). Thus, for every *z* ∈ *x* + *y*, we have *z* ∈ *T* and so it follows that *x* + *y*⊆*T*. As such, for every *z* ∈ *T*, we have *z* + *T*⊆*T*.

Now let *x*, *z* ∈ *T*. Then $t_{{\left({\hat{F}}_{a}\right)}_{T}}\left(x\right)=t_{{\left({\hat{F}}_{a}\right)}_{T}}\left(z\right)=s$ and $f_{{\left({\hat{F}}_{a}\right)}_{T}}\left(x\right)=f_{{\left({\hat{F}}_{a}\right)}_{T}}\left(z\right)=w$ and so ${\left({\hat{F}}_{a}\right)}_{T}\left(x\right)={\left({\hat{F}}_{a}\right)}_{T}\left(z\right)=s$. Since ${\hat{F}}_{a}$ is a vague subhypergroup of (*R*, +) for all $a\in Supp\left(\hat{F},A\right)$, there exists *y* ∈ *R* such that *x* ∈ *z* + *y* and $\min\left\{{\left({\hat{F}}_{a}\right)}_{T}\left(x\right),{\left({\hat{F}}_{a}\right)}_{T}\left(z\right)\right\}\leq {\left({\hat{F}}_{a}\right)}_{T}\left(y\right)$. Thus we obtain $\min\left\{{\left({\hat{F}}_{a}\right)}_{T}\left(x\right),{\left({\hat{F}}_{a}\right)}_{T}\left(z\right)\right\}=\min\left\{s,s\right\}=s$ and therefore ${\left({\hat{F}}_{a}\right)}_{T}(y)\geq s$ which implies that *y* ∈ *T*. Since *x*, *y* ∈ *T*, this implies that *T*⊆*z* + *T* and so we have *z* + *T* = *T* for all *z* ∈ *T*. Therefore it has been proven that (*T*, +) is a subhypergroup of (*R*, +). Let *x*, *y* ∈ *T*. Then $t_{{\left({\hat{F}}_{a}\right)}_{T}}\left(x\right)=t_{{\left({\hat{F}}_{a}\right)}_{T}}\left(y\right)=s$ and $f_{{\left({\hat{F}}_{a}\right)}_{T}}\left(x\right)=f_{{\left({\hat{F}}_{a}\right)}_{T}}\left(y\right)=w$ and thus ${\left({\hat{F}}_{a}\right)}_{T}\left(x\right)={\left({\hat{F}}_{a}\right)}_{T}\left(y\right)=s$. Now since (*R*, ∘) is a semihypergroup, ${\hat{F}}_{a}$ is a vague subhypergroup of (*R*, ∘) for all $a\in Supp\left(\hat{F},A\right)$. Thus it follows that $\min\left\{{\left({\hat{F}}_{a}\right)}_{T}\left(x\right),{\left({\hat{F}}_{a}\right)}_{T}\left(y\right)\right\}=\min\left\{s,s\right\}=s$ and therefore $\inf\left\{{\left({\hat{F}}_{a}\right)}_{T}\left(z\right):z\in x\circ y\right\}\geq s$ too. As a result, for every *z* ∈ *x*∘*y*, we have *z* ∈ *T* and therefore *x*∘*y*⊆*T*. As such, for every *x*, *y* ∈ *T*, we have *x*∘*y* ∈ *P* ^*∗*^(*T*). Hence *T* is a subhyperring of *R*.

**Theorem 32 .**

Let *T* be a nonempty subset of *R*, let $\left(\hat{F},A\right)$ be a nonnull vague soft set over *R*, and let ${\left(\hat{F},A\right)}_{T}$ be a vague soft characteristic set over *T*. Then $\left(\hat{F},A\right)$ is a vague soft hyperring over *R* if and only if *T* is a subhyperring of *R*.

**Proof**

The proof is similar to that of Theorem 31.

Theorem 32 implies that there exists a one-to-one correspondence between vague soft hyperrings and classical subhyperrings of a hyperring.

## 4. Vague Soft Hyperideals 

The ideal of a ring is an important concept in the study of rings. The concept of a fuzzy ideal of a ring was introduced by Liu (see [9]). Davvaz (see [8]) introduced the notion of a fuzzy hyperideal (resp., fuzzy *H*_*ν*_-ideal) of a hyperring (resp., *H*_*ν*_-ring). In this section the notion of vague soft hyperideals of a hyperring is defined using Rosenfeld's approach.

**Definition 33 .**

Let (*F*, *A*) be a nonnull soft set over *R*. Then (*F*, *A*) is called a* soft left* (resp.,* right*)* hyperideal *of *R* if *F*(*x*) is a left (resp., right) hyperideal of *R* for all $x\in Supp\left(\hat{F},A\right)$.

**Definition 34 .**

Let (*F*, *A*) be a nonnull soft set over *R*. Then (*F*, *A*) is called a* soft hyperideal* of *R* if *F*(*x*) is a hyperideal of *R*; that is, *F*(*x*) is both right and left hyperideals of *R* for all $x\in Supp\left(\hat{F},A\right)$.

**Definition 35 .**

Let $\left(\hat{F},A\right)$ be a nonnull vague soft set over *R*. Then $\left(\hat{F},A\right)$ is called a* vague soft left* (resp.,* right*)* hyperideal* of *R* if the following axioms hold for all $a\in Supp\left(\hat{F},A\right)$:

- for all *x*, *y* ∈ *R*, $\min\left\{t_{{\hat{F}}_{a}}\left(x\right),t_{{\hat{F}}_{a}}\left(y\right)\right\}\leq \inf\left\{t_{{\hat{F}}_{a}}\left(z\right):z\in x+y\right\}$ and $\min\left\{{1-f}_{{\hat{F}}_{a}}\left(x\right),{1-f}_{{\hat{F}}_{a}}\left(y\right)\right\}\leq \inf\left\{{1-f}_{{\hat{F}}_{a}}\left(z\right):z\in x+y\right\}$, which means that $\min\left\{{\hat{F}}_{a}\left(x\right),{\hat{F}}_{a}\left(y\right)\right\}\leq \inf\left\{{\hat{F}}_{a}\left(z\right):z\in x+y\right\}$,
- for all *w*, *x* ∈ *R*, there exists *y* ∈ *R* such that *x* ∈ *w* + *y* and $\min\left\{t_{{\hat{F}}_{a}}\left(w\right),t_{{\hat{F}}_{a}}\left(x\right)\right\}\leq t_{{\hat{F}}_{a}}(y)$ and $\min\left\{{1-f}_{{\hat{F}}_{a}}\left(w\right),{1-f}_{{\hat{F}}_{a}}\left(x\right)\right\}\leq {1-f}_{{\hat{F}}_{a}}\left(y\right)$; that is, $\min\left\{{\hat{F}}_{a}\left(w\right),{\hat{F}}_{a}\left(x\right)\right\}\leq \;{\hat{F}}_{a}\left(y\right)$,
- for all *w*, *x* ∈ *R*, there exists *z* ∈ *R* such that *x* ∈ *z* + *w* and $\min\left\{t_{{\hat{F}}_{a}}\left(w\right),t_{{\hat{F}}_{a}}\left(x\right)\right\}\leq t_{{\hat{F}}_{a}}(z)$ and $\min\left\{{1-f}_{{\hat{F}}_{a}}\left(w\right),{1-f}_{{\hat{F}}_{a}}\left(x\right)\right\}\leq {1-f}_{{\hat{F}}_{a}}\left(z\right)$; that is, $\min\left\{{\hat{F}}_{a}\left(w\right),{\hat{F}}_{a}\left(x\right)\right\}\leq \;{\hat{F}}_{a}\left(z\right)$,
- for all *x*, *y* ∈ *R*, $t_{{\hat{F}}_{a}}\left(y\right)\leq \inf\left\{t_{{\hat{F}}_{a}}\left(z\right):z\in x\circ y\right\}$ (resp., $t_{{\hat{F}}_{a}}\left(x\right)\leq \inf\{t_{{\hat{F}}_{a}}\left(z\right):z\in x\circ y\}$) and ${1-f}_{{\hat{F}}_{a}}\left(y\right)\leq \inf\left\{{1-f}_{{\hat{F}}_{a}}\left(z\right):z\in x\circ y\right\}$ (resp., ${1-f}_{{\hat{F}}_{a}}\left(x\right)\leq \inf\left\{{1-f}_{{\hat{F}}_{a}}\left(z\right):z\in x\circ y\right\}$); that is, ${\hat{F}}_{a}\left(y\right)\leq \inf\left\{{\hat{F}}_{a}\left(z\right):z\in x\circ y\right\}$ (resp., ${\hat{F}}_{a}\left(x\right)\leq \inf\left\{{\hat{F}}_{a}\left(z\right):z\in x\circ y\right\}$).

That is, ${\hat{F}}_{a}$ is a vague left (resp., right) hyperideal of *R* (in Rosenfeld's sense) for all $a\in Supp\left(\hat{F},A\right)$.

Conditions (i), (ii), and (iii) prove that ${\hat{F}}_{a}$ is a vague subhypergroup of (*R*, +). This means that $\left(\hat{F},A\right)$ is a* vague soft left* (resp.,* right*)* hyperideal* of *R* if, for all $a\in Supp\left(\hat{F},A\right)$, ${\hat{F}}_{a}$ is a vague subhypergroup of (*R*, +) and ${\hat{F}}_{a}$ also satisfies the condition ${\hat{F}}_{a}\left(y\right)\leq \inf\left\{{\hat{F}}_{a}\left(z\right):z\in x\circ y\right\}$ (resp., ${\hat{F}}_{a}\left(x\right)\leq \inf\left\{{\hat{F}}_{a}\left(z\right):z\in x\circ y\right\}$).

**Definition 36 .**

Let $\left(\hat{F},A\right)$ be a nonnull vague soft set over *R*. Then $\left(\hat{F},A\right)$ is called a* vague soft hyperideal* of *R* if the following axioms hold for all $a\in Supp\left(\hat{F},A\right)$:

- for all *x*, *y* ∈ *R*, $\min\left\{t_{{\hat{F}}_{a}}\left(x\right),t_{{\hat{F}}_{a}}\left(y\right)\right\}\leq \inf\left\{t_{{\hat{F}}_{a}}\left(z\right):z\in x+y\right\}$ and $\min\left\{{1-f}_{{\hat{F}}_{a}}\left(x\right),{1-f}_{{\hat{F}}_{a}}\left(y\right)\right\}\leq \inf\left\{{1-f}_{{\hat{F}}_{a}}\left(z\right):z\in x+y\right\}$, which means that $\min\left\{{\hat{F}}_{a}\left(x\right),{\hat{F}}_{a}\left(y\right)\right\}\leq \inf\left\{{\hat{F}}_{a}\left(z\right):z\in x+y\right\}$,
- for all *w*, *x* ∈ *R*, there exists *y* ∈ *R* such that *x* ∈ *w* + *y* and $\min\left\{t_{{\hat{F}}_{a}}\left(w\right),t_{{\hat{F}}_{a}}\left(x\right)\right\}\leq t_{{\hat{F}}_{a}}(y)$ and $\min\left\{{1-f}_{{\hat{F}}_{a}}\left(w\right),{1-f}_{{\hat{F}}_{a}}\left(x\right)\right\}\leq {1-f}_{{\hat{F}}_{a}}\left(y\right)$; that is, $\min\left\{{\hat{F}}_{a}\left(w\right),{\hat{F}}_{a}\left(x\right)\right\}\leq \;{\hat{F}}_{a}\left(y\right)$,
- for all *w*, *x* ∈ *R*, there exists *z* ∈ *R* such that *x* ∈ *z* + *w* and $\min\left\{t_{{\hat{F}}_{a}}\left(w\right),t_{{\hat{F}}_{a}}\left(x\right)\right\}\leq t_{{\hat{F}}_{a}}(z)$ and $\min\left\{{1-f}_{{\hat{F}}_{a}}\left(w\right),{1-f}_{{\hat{F}}_{a}}\left(x\right)\right\}\leq {1-f}_{{\hat{F}}_{a}}\left(z\right)$; that is, $\min\left\{{\hat{F}}_{a}\left(w\right),{\hat{F}}_{a}\left(x\right)\right\}\leq \;{\hat{F}}_{a}\left(z\right)$,
- for all *x*, *y* ∈ *R*, $\max\left\{t_{{\hat{F}}_{a}}\left(x\right),t_{{\hat{F}}_{a}}\left(y\right)\right\}\leq \inf\left\{t_{{\hat{F}}_{a}}\left(z\right):z\in x\circ y\right\}$ and $\max\left\{{1-f}_{{\hat{F}}_{a}}\left(x\right),{1-f}_{{\hat{F}}_{a}}\left(y\right)\right\}\leq \inf\left\{{1-f}_{{\hat{F}}_{a}}\left(z\right):z\in x\circ y\right\}$; that is, $\max\left\{{\hat{F}}_{a}\left(x\right),{\hat{F}}_{a}\left(y\right)\right\}\leq \inf\left\{{\hat{F}}_{a}\left(z\right):z\in x\circ y\right\}$.

That is, ${\hat{F}}_{a}$ is a* vague hyperideal* of *R* (in Rosenfeld's sense) for all $a\in Supp\left(\hat{F},A\right)$.

Similar to Definition 35, conditions (i), (ii), and (iii) of Definition 36 indicate that ${\hat{F}}_{a}$ is a vague subhypergroup of (*R*, +) while condition (iv) is a combination of both the conditions given in Definition 35 (condition (iv)).

**Theorem 37 .**

Let $\left(\hat{F},A\right)$ be a nonnull vague soft set over *R*. Then the necessary and sufficient condition for $\left(\hat{F},A\right)$ to be a vague soft hyperideal is for $\left(\hat{F},A\right)$ to be a vague soft hypergroup of (*R*, +) and $\left(\hat{F},A\right)$ is both a vague soft left hyperideal and a vague soft right hyperideal of *R*.

**Proof**

The proof is straightforward by Definitions 35 and 36.

**Proposition 38 .**

Let $\left(\hat{F},A\right)$ and $\left(\hat{G},B\right)$ be two vague soft hyperideals of *R*. Then $\left(\hat{F},A\right)\;\tilde{\wedge }\;(\hat{G},B)$ is a vague soft hyperideal of *R* if it is nonnull.

**Proof**

Suppose that $\left(\hat{F},A\right)$ and $\left(\hat{G},B\right)$ are vague soft hyperideals of *R*. Now to prove that $\left(\hat{H},A\times B\right)$ is a vague soft hyperideal of *R*, we have to prove that all the conditions of Definition 36 have been satisfied. However, in Proposition 27, it has been proven that conditions (i), (ii), and (iii) have been satisfied. Therefore it is only necessary to prove that $\left(\hat{H},A\times B\right)$ satisfies condition (iv) of Definition 36.

Thus for all *x*, *y* ∈ *R* and $\left(\alpha ,\beta \right)\in Supp\left(\hat{H},A\times B\right)$ we have

(21) max⁡tH^α,βx,tH^α,βy=max⁡tF^αx∩tG^βx,tF^αy∩tG^βy≤max⁡tF^αx,tF^αy∩max⁡tG^βx,tG^βy≤inf⁡tF^αz:z∈x∘y∩inf⁡tG^βz:z∈x∘y≤inf⁡tF^αz∩tG^βz:z∈x∘y=inf⁡tH^α,β:z∈x∘y.

Similarly, it can be proven that the condition $\max\left\{{1-f}_{{\hat{H}}_{\left(\alpha ,\beta \right)}}\left(x\right),1-f_{{\hat{H}}_{\left(\alpha ,\beta \right)}}\left(y\right)\right\}\leq \inf\left\{{1-f}_{{\hat{H}}_{\left(\alpha ,\beta \right)}}:z\in x\circ y\right\}$ is satisfied. This means that $\max\left\{{\hat{H}}_{\left(\alpha ,\beta \right)}\left(x\right),{\hat{H}}_{\left(\alpha ,\beta \right)}\left(y\right)\right\}\leq \inf\left\{{\hat{H}}_{\left(\alpha ,\beta \right)}\left(z\right):z\in x\circ y\right\}$. Thus it has been proven that ${\hat{H}}_{\left(\alpha ,\beta \right)}$ is a vague hyperideal of *R* for all $\left(\alpha ,\beta \right)\in Supp\left(\hat{H},A\times B\right)$ and as such $\left(\hat{H},A\times B\right)=\left(\hat{F},A\right)\;\tilde{\wedge }\;(\hat{G},B)$ is a vague soft hyperideal of *R*.

**Theorem 39 .**

Let $\left(\hat{F},A\right)$ be a nonnull vague soft set over *R*. Then $\left(\hat{F},A\right)$ is a vague soft hyperideal of *R* if and only if, for each *t* ∈ [0, 1], ${\left(\hat{F},A\right)}_{\left(t,t\right)}$ is a soft hyperideal of *R*.

**Proof**

(⇒) Let $\left(\hat{F},A\right)$ be a vague soft hyperideal of *R* and *t* ∈ [0, 1]. Then ${\hat{F}}_{a}$ is a vague subhypergroup of (*R*, +). Now let $x,y\in {\left({\hat{F}}_{a}\right)}_{\left(t,t\right)}$. Then $t_{{\hat{F}}_{a}}\left(x\right),t_{{\hat{F}}_{a}}(y)\geq t$ and ${1-f}_{{\hat{F}}_{a}}\left(x\right),{1-f}_{{\hat{F}}_{a}}\left(y\right)\geq t$, which means that ${\hat{F}}_{a}(x)\geq t$ and ${\hat{F}}_{a}\left(y\right)\geq t$. In Theorem 28, it was proved that if ${\hat{F}}_{a}$ is a vague subhypergroup of (*R*, +), then ${\left({\hat{F}}_{a}\right)}_{\left(t,t\right)}$ is a subhypergroup of (*R*, +). This means that it is only necessary to prove that ${\left({\hat{F}}_{a}\right)}_{\left(t,t\right)}$ satisfies the following conditions:

(22) R∘F^at,t⊆F^at,t,F^at,t∘R⊆F^at,t.

Now let *x* ∈ *R* and $y\in {\left({\hat{F}}_{a}\right)}_{\left(t,t\right)}$. Then $t_{{\hat{F}}_{a}}(y)\geq t$ and ${1-f}_{{\hat{F}}_{a}}(y)\geq t$ which means that ${\hat{F}}_{a}\left(y\right)\geq t$. Since ${\hat{F}}_{a}$ is a vague hyperideal of *R* for all $a\in Supp\left(\hat{F},A\right)$, ${\hat{F}}_{a}$ must satisfy the following condition:

(23) tF^ay≤inf⁡tF^az:z∈x∘y,1−fF^ay≤inf⁡1−fF^az:z∈x∘y,

which means ${\hat{F}}_{a}\left(y\right)\leq \inf\left\{{\hat{F}}_{a}\left(z\right):z\in x\circ y\right\}$. Therefore we obtain $\inf\left\{{\hat{F}}_{a}\left(z\right):z\in x\circ y\right\}\geq {\hat{F}}_{a}\left(y\right)\geq t$. This proves that $z\in {\left({\hat{F}}_{a}\right)}_{\left(t,t\right)}$ for all *z* ∈ *x*∘*y* and as a result we obtain $x\circ y\subseteq {\left({\hat{F}}_{a}\right)}_{\left(t,t\right)}$. Thus for every *x* ∈ *R* and $y\in {\left({\hat{F}}_{a}\right)}_{\left(t,t\right)}$, we obtain $R\circ {\left({\hat{F}}_{a}\right)}_{\left(t,t\right)}\subseteq {\left({\hat{F}}_{a}\right)}_{\left(t,t\right)}$ and this proves that ${\left({\hat{F}}_{a}\right)}_{\left(t,t\right)}$ is a left hyperideal of *R*. This implies that ${\left(\hat{F},A\right)}_{\left(t,t\right)}$ is a soft left hyperideal of *R*. Similarly, it can been proven that ${\left(\hat{F},A\right)}_{\left(t,t\right)}$ is a soft right hyperideal of *R*. Thus ${\left(\hat{F},A\right)}_{\left(t,t\right)}$ is a soft right hyperideal of *R*. Hence ${\left(\hat{F},A\right)}_{\left(t,t\right)}$ is a soft left hyperideal of *R* and a soft right hyperideal of *R* which proves that ${\left(\hat{F},A\right)}_{\left(t,t\right)}$ is a soft hyperideal of *R*.

(⇐) Let ${\left(\hat{F},A\right)}_{\left(t,t\right)}$ be a soft hyperideal of *R*. Then, for each $a\in Supp\left(\hat{F},A\right)$, ${\left({\hat{F}}_{a}\right)}_{\left(t,t\right)}$ is a nonnull hyperideal of *R*. Therefore by Definition 16, ${\left({\hat{F}}_{a}\right)}_{\left(t,t\right)}$ is a subhypergroup of (*R*, +). This implies that ${\left(\hat{F},A\right)}_{\left(t,t\right)}$ is a soft hypergroup over (*R*, +). In [10], it was proven that if ${\left(\hat{F},A\right)}_{\left(t,t\right)}$ is a soft hypergroup, then $\left(\hat{F},A\right)$ is a vague soft hypergroup. Thus it can be concluded that $\left(\hat{F},A\right)$ is a vague soft hypergroup over (*R*, +). Furthermore, since ${\left({\hat{F}}_{a}\right)}_{\left(t,t\right)}$ is both a left hyperideal and a right hyperideal of *R*, the following conditions are satisfied:

(24) R∘F^at,t⊆F^at,t,F^at,t∘R⊆F^at,t.

Now let *x* ∈ *R* and $y\in {\left({\hat{F}}_{a}\right)}_{\left(t,t\right)}$ or $x\in {\left({\hat{F}}_{a}\right)}_{\left(t,t\right)}$ and *y* ∈ *R*. Thus we obtain $x\circ y\subseteq {\left({\hat{F}}_{a}\right)}_{\left(t,t\right)}$ and $y\circ x\subseteq {\left({\hat{F}}_{a}\right)}_{\left(t,t\right)}$ which implies that *z* ∈ *x*∘*y*, where $z\in {\left({\hat{F}}_{a}\right)}_{\left(t,t\right)}$. As such, we obtain $\inf\left\{{\hat{F}}_{a}\left(z\right):z\in x\circ y\right\}\geq t$. Now let $x,y\in {\left({\hat{F}}_{a}\right)}_{\left(t,t\right)}$. Then ${\hat{F}}_{a}(x)\geq t$ and ${\hat{F}}_{a}\left(y\right)\geq t$. Therefore, we obtain $\inf\left\{{\hat{F}}_{a}\left(z\right):z\in x\circ y\right\}\geq {\hat{F}}_{a}(x)\geq t$ as well as $\inf\left\{{\hat{F}}_{a}\left(z\right):z\in x\circ y\right\}\geq {\hat{F}}_{a}(y)\geq t$ which means that ${\hat{F}}_{a}(x)\leq \inf\left\{{\hat{F}}_{a}\left(z\right):z\in x\circ y\right\}$ and ${\hat{F}}_{a}\left(y\right)\leq \inf\left\{{\hat{F}}_{a}\left(z\right):z\in x\circ y\right\}$. Combining these conditions, we obtain $\max\left\{{\hat{F}}_{a}\left(x\right),{\hat{F}}_{a}\left(y\right)\right\}\leq \inf\left\{{\hat{F}}_{a}\left(z\right):z\in x\circ y\right\}$. Thus condition (iv) of Definition 36 has also been verified. This means that ${\hat{F}}_{a}$ is a vague hyperideal of *R* and hence it proves that $\left(\hat{F},A\right)$ is a vague soft hyperideal of *R*.

Theorem 39 proves that there exists a one-to-one correspondence between vague soft hyperideal and the soft hyperideal of a hyperring.

**Theorem 40 .**

Let $\left(\hat{F},A\right)$ be a nonnull vague soft set over *R* left (resp., right) hyperideal of *R*. Then, for all *α*, *β* ∈ [0, 1], ${\left(\hat{F},A\right)}_{\left(\alpha ,\beta \right)}$ is a left (resp., right) hyperideal of *R*.

**Proof**

Let $\left(\hat{F},A\right)$ be a vague soft left (resp., right) hyperideal of *R*. Then, by Definition 35, $\left(\hat{F},A\right)$ is a vague soft hypergroup over (*R*, +). Now in Theorem 29 it was proven that if $\left(\hat{F},A\right)$ is a vague soft hypergroup over (*R*, +), then ${\left(\hat{F},A\right)}_{\left(\alpha ,\beta \right)}$ is a subhypergroup of (*R*, +). Therefore, in order to prove that ${\left(\hat{F},A\right)}_{\left(\alpha ,\beta \right)}$ is a left (resp., right) hyperideal of *R*, we only have to prove that $R\circ {\left(\hat{F},A\right)}_{\left(\alpha ,\beta \right)}\subseteq {\left(\hat{F},A\right)}_{\left(\alpha ,\beta \right)}$ (resp., ${\left(\hat{F},A\right)}_{\left(\alpha ,\beta \right)}\circ R\subseteq {\left(\hat{F},A\right)}_{\left(\alpha ,\beta \right)}$). Now let *x* ∈ *R* and $y\in {\left(\hat{F},A\right)}_{\left(\alpha ,\beta \right)}$. Then $t_{{\hat{F}}_{a}}(y)\geq \alpha$ and ${1-f}_{{\hat{F}}_{a}}(y)\geq \beta$. Since ${\hat{F}}_{a}$ is a vague left ideal of *R* for all $a\in Supp\left(\hat{F},A\right)$, ${\hat{F}}_{a}$ satisfies the conditions $t_{{\hat{F}}_{a}}(y)\leq \inf\left\{t_{{\hat{F}}_{a}}\left(z\right):z\in x\circ y\right\}$ and ${1-f}_{{\hat{F}}_{a}}\left(y\right)\leq \inf\left\{{1-f}_{{\hat{F}}_{a}}\left(z\right):z\in x\circ y\right\}$. Therefore we obtain $\inf\left\{t_{{\hat{F}}_{a}}\left(z\right):z\in x\circ y\right\}\geq t_{{\hat{F}}_{a}}(y)\geq \alpha$ and $\inf\left\{{1-f}_{{\hat{F}}_{a}}\left(z\right):z\in x\circ y\right\}\geq {1-f}_{{\hat{F}}_{a}}\left(y\right)\geq \beta$ which implies that, for all *z* ∈ *x*∘*y*, $z\in {\left(\hat{F},A\right)}_{\left(\alpha ,\beta \right)}$. As such, we obtain $x\circ y\subseteq {\left(\hat{F},A\right)}_{\left(\alpha ,\beta \right)}$. Thus for every *x* ∈ *R* and $y\in {\left(\hat{F},A\right)}_{\left(\alpha ,\beta \right)}$, we obtain $R\circ {\left(\hat{F},A\right)}_{\left(\alpha ,\beta \right)}\subseteq {\left(\hat{F},A\right)}_{\left(\alpha ,\beta \right)}$. Hence ${\left(\hat{F},A\right)}_{\left(\alpha ,\beta \right)}$ is a left hyperideal of *R*. Next let $x\in {\left(\hat{F},A\right)}_{\left(\alpha ,\beta \right)}$ and *y* ∈ *R*. Then $t_{{\hat{F}}_{a}}(x)\geq \alpha$ and ${1-f}_{{\hat{F}}_{a}}\left(x\right)\geq \beta$. Similarly, we obtain $\inf\left\{t_{{\hat{F}}_{a}}\left(z\right):z\in x\circ y\right\}\geq t_{{\hat{F}}_{a}}(x)\geq \alpha$ and $\inf\left\{{1-f}_{{\hat{F}}_{a}}\left(z\right):z\in x\circ y\right\}\geq {1-f}_{{\hat{F}}_{a}}\left(x\right)\geq \beta$. This implies that $z\in {\left(\hat{F},A\right)}_{\left(\alpha ,\beta \right)}$ for all *z* ∈ *x*∘*y* and therefore we obtain $x\circ y\subseteq {\left(\hat{F},A\right)}_{\left(\alpha ,\beta \right)}$. As such, for every $x\in {\left(\hat{F},A\right)}_{\left(\alpha ,\beta \right)}$ and *y* ∈ *R*, we obtain ${\left(\hat{F},A\right)}_{\left(\alpha ,\beta \right)}\circ R\subseteq {\left(\hat{F},A\right)}_{\left(\alpha ,\beta \right)}$ which means ${\left(\hat{F},A\right)}_{\left(\alpha ,\beta \right)}$ is a right hyperideal of *R*.

**Theorem 41 .**

Let $\left(\hat{F},A\right)$ be a nonnull vague soft set over *R*. Then $\left(\hat{F},A\right)$ is a vague soft left (resp., right) hyperideal of *R* if and only if, for all *α*, *β* ∈ [0, 1], ${\left(\hat{F},A\right)}_{\left(\alpha ,\beta \right)}$ is a left (resp., right) hyperideal of *R*.

**Proof**

The proof is straightforward.

**Theorem 42 .**

Let *M* be a nonempty subset of *R* and ${\left(\hat{F},A\right)}_{M}$ be a vague soft characteristic set over *M*. If $\left(\hat{F},A\right)$ is a vague soft hyperideal of *R*, then *M* is a hyperideal of *R*.

**Proof**

Let $\left(\hat{F},A\right)$ be a vague soft hyperideal of *R*. Therefore, by Definition 36, $\left(\hat{F},A\right)$ is a vague soft hypergroup over (*R*, +) and satisfies the condition $\max\left\{{\hat{F}}_{a}\left(x\right),{\hat{F}}_{a}\left(y\right)\right\}\leq \inf\left\{{\hat{F}}_{a}\left(z\right):z\in x\circ y\right\}$. In order to prove that *M* is a hyperideal of *R*, we need to prove that (*M*, +) is a subhypergroup of (*R*, +) and *R*∘*M*⊆*M* and *M*∘*R*⊆*M*. In Theorem 31, it was proven that (*M*, +) is a subhypergroup of (*R*, +) if $\left(\hat{F},A\right)$ is a vague soft hypergroup over (*R*, +). Now let *x* ∈ *R* and *y* ∈ *M*. Then $t_{{\hat{F}}_{a}}\left(y\right)={1-f}_{{\hat{F}}_{a}}\left(y\right)=s$ which means that ${\hat{F}}_{a}\left(y\right)=s$. Since ${\hat{F}}_{a}$ is a vague hyperideal of *R* for all $a\in Supp\left(\hat{F},A\right)$, it must satisfy the following conditions:

(25) F^ay≤inf⁡F^az:z∈x∘y,F^ax≤inf⁡F^az:z∈x∘y.

Therefore since ${\hat{F}}_{a}\left(y\right)=s$ for all $a\in Supp\left(\hat{F},A\right)$ and *y* ∈ *M*, we obtain $\inf\left\{{\hat{F}}_{a}\left(z\right):z\in x\circ y\right\}\geq {\hat{F}}_{a}\left(y\right)=s$ which means that $\inf\left\{{\hat{F}}_{a}\left(z\right):z\in x\circ y\right\}\geq s$. This implies that *z* ∈ *M* for all *z* ∈ *x*∘*y* and this proves that *x*∘*y*⊆*M* for all *x* ∈ *R* and *y* ∈ *M*. Therefore we obtain *R*∘*M*⊆*M* and this implies that *M* is a left hyperideal of *R*. Now let *x* ∈ *M* and *y* ∈ *R*. Then $t_{{\hat{F}}_{a}}\left(x\right)=1-f_{{\hat{F}}_{a}}=s$ which means that ${\hat{F}}_{a}\left(x\right)=s$. Similarly, we obtain $\inf\left\{{\hat{F}}_{a}\left(z\right):z\in x\circ y\right\}\geq {\hat{F}}_{a}\left(x\right)=s$ which means that $\inf\left\{{\hat{F}}_{a}\left(z\right):z\in x\circ y\right\}\geq s$. This implies that *z* ∈ *M* for all *z* ∈ *x*∘*y* and consequently we obtain *x*∘*y*⊆*M* for all *x* ∈ *M* and *y* ∈ *R*. This proves that *M*∘*R*⊆*M*. As such, *M* is a right hyperideal of *R*. Hence it has been proven that *M* is a hyperideal of *R*.

**Theorem 43 .**

Let *M* be a nonempty subset of *R*, let $\left(\hat{F},A\right)$ be a nonnull vague soft set over *R*, and let ${\left(\hat{F},A\right)}_{M}$ be a vague soft characteristic set over *M*. Then $\left(\hat{F},A\right)$ is a vague soft hyperideal of *R* if and only if *M* is a hyperideal of *R*.

**Proof**

The proof is straightforward.

## 5. Vague Soft Hyperring Homomorphism 

In this section, we introduce the notion of vague soft hyperring homomorphism by applying the concept of vague soft functions as well as the notion of the image and preimage of a vague soft set under a vague soft function introduced by [10] in the context of vague soft hyperrings. Lastly, it is proven that the vague soft hyperring homomorphism preserves vague soft hyperrings.

**Definition 44 (see [2]).**

Let (*R*_1_, +_1_, ∘_1_) and (*R*_2_, +_2_, ∘_2_) be two hyperrings. A map *f* : *R*_1_ → *R*_2_ is called a* hyperring homomorphism* if, for all *x*, *y* ∈ *R*_1_, one has *f*(*x*  +_1_ *y*)⊆*f*(*x*)  +_2_ *f*(*y*) and *f*(*x* ∘_1_ *y*)⊆*f*(*x*) ∘_2_ *f*(*y*).

**Definition 45 (see [10]).**

Let *φ* : *X* → *Y* and *ψ* : *A* → *B* be two functions, where *A* and *B* are parameter sets for the classical sets *X* and *Y*, respectively. Let $\left(\hat{F},A\right)$ and $\left(\hat{G},B\right)$ be vague soft sets over *X* and *Y*, respectively. Then the ordered pair (*φ*, *ψ*) is called a* vague soft function* from $\left(\hat{F},A\right)$ to $\left(\hat{G},B\right)$ and is denoted by $\left(\varphi ,\psi \right):\left(\hat{F},A\right)\to \left(\hat{G},B\right)$.

**Definition 46 (see [10]).**

Let $\left(\hat{F},A\right)$ and $\left(\hat{G},B\right)$ be vague soft sets over *X* and *Y*, respectively. Let $\left(\varphi ,\psi \right):\left(\hat{F},A\right)\to (\hat{G},B)$ be a vague soft function.

(i) The* image* of $\left(\hat{F},A\right)$ under the vague soft function (*φ*, *ψ*), which is denoted by $\left(\varphi ,\psi \right)\left(\hat{F},A\right)$, is a vague soft set over *Y*, which is defined as
  (26) $$\begin{array}{c} \left(\;\varphi ,\psi \;\right)\left(\;\hat{F},A\;\right)=\left(\;\varphi \left(\;\hat{F}\;\right),\psi \left(\;A\;\right)\;\right), \end{array}$$
 where
  (27) $$\begin{array}{c} \varphi \left(\;{\hat{F}}_{a}\;\right)\left(\;\varphi \left(\;x\;\right)\;\right)={\left(\;\varphi \left(\;\hat{F}\;\right)\;\right)}_{\psi \left(\;a\;\right)}\left(\;y\;\right), \end{array}$$
 for all *a* ∈ *A*, *x* ∈ *X*, and *y* ∈ *Y*.
(ii) The* preimage* of $\left(\hat{G},B\right)$ under the vague soft function (*φ*, *ψ*), which is denoted by ${\left(\varphi ,\psi \right)}^{-1}\left(\hat{G},B\right)$, is a vague soft set over *X*, which is defined as
  (28) $$\begin{array}{c} {\left(\;\varphi ,\psi \;\right)}^{-1}\left(\;\hat{G},B\;\right)=\left(\;\varphi^{-1}\left(\;\hat{G}\;\right),\psi^{-1}\left(\;B\;\right)\;\right), \end{array}$$
 where
  (29) $$\begin{array}{c} \varphi^{-1}\left(\;{\hat{G}}_{b}\;\right)\left(\;\varphi^{-1}\left(\;y\;\right)\;\right)={\left(\;\varphi^{-1}\left(\;\hat{G}\;\right)\;\right)}_{\psi^{-1}\left(\;b\;\right)}\left(\;x\;\right), \end{array}$$
 for all *b* ∈ *B*, *x* ∈ *X*, and *y* ∈ *Y*.

If *φ* and *ψ* are injective (surjective), then the vague soft function (*φ*, *ψ*) is said to be injective (surjective).

**Definition 47 .**

Let (*R*_1_, +_1_, ∘_1_) and (*R*_2_, +_2_, ∘_2_) be two hyperrings. Also let $\left(\hat{F},A\right)$ and $\left(\hat{G},B\right)$ be two vague soft hyperrings over *R*_1_ and *R*_2_, respectively, and let $\left(\varphi ,\psi \right):(\hat{F},A)\to \left(\hat{G},B\right)$ be a vague soft function. Then $\left(\varphi ,\psi \right):(\hat{F},A)\to \left(\hat{G},B\right)$ is called a* vague soft hyperring homomorphism* if the following conditions are satisfied:

- *φ* is a hyperring homomorphism from *R*_1_ to *R*_2_,
- *ψ* is a function from *A* to *B*,
- $\varphi \left(\hat{F}\left(x\right)\right)=\hat{G}\left(\psi \left(x\right)\right)$ for all $x\in Supp\left(\hat{F},A\right)$.

**Theorem 48 .**

Let (*R*_1_, +_1_, ∘_1_) and (*R*_2_, +_2_, ∘_2_) be two hyperrings. Let $\left(\hat{F},A\right)$ and $\left(\hat{G},B\right)$ be two vague soft hyperrings over *R*_1_ and *R*_2_, respectively. Let $\left(\varphi ,\psi \right):(\hat{F},A)\to \left(\hat{G},B\right)$ be a vague soft hyperring homomorphism and let *ψ* : *A* → *B* be an injective mapping. Then $\left(\varphi \left(\hat{F}\right),\psi \left(A\right)\right)$ is a vague soft hyperring over (*R*_2_, +_2_, ∘_2_).

**Proof**

Since (*φ*, *ψ*) is a vague soft hyperring homomorphism, it can be seen that

(30) $$\begin{array}{c} Supp\left(\;\varphi \left(\;\hat{F}\;\right),\psi \left(\;A\;\right)\;\right)\subseteq \psi \left(\;Supp\left(\;\hat{F},A\;\right)\;\right). \end{array}$$

Now let *w*_1_, *w*_2_ ∈ *R*_2_. Then there exists *x*, *y* ∈ *R*_1_ such that *φ*(*x*) = *w*_1_ and *φ*(*y*) = *w*_2_. Therefore, for all *x*, *y* ∈ *R*_1_, $a\in Supp\left(\hat{F},A\right)$ we obtain

(31) min⁡φF^aφx,φF^aφy=min⁡φF^ψaw1,φF^ψaw2≤inf⁡φF^ψau:u∈w1  +2  w2=inf⁡φF^aφz,

where *z* ∈ *R*_1_ such that *z* ∈ *x*  +_1_ *y* and *φ*(*z*) = *u*.

Furthermore, for all *w*, *x* ∈ *R*_1_, there exists *u*_1_, *u*_2_ ∈ *R*_2_ such that *φ*(*w*) = *u*_1_ and *φ*(*x*) = *u*_2_. Therefore, for all *w*, *x* ∈ *R*_1_ and for each $a\in Supp\left(\hat{F},A\right)$, we obtain

(32) min⁡φF^aφw,φF^aφx=min⁡φF^ψau1,φF^ψau2≤inf⁡φF^ψab:u2∈u1  +2  b=inf⁡φF^aφy,

where *y* ∈ *R*_1_ such that *x* ∈ *w*  +_1_ *y* and *φ*(*y*) = *b* and also

(33) min⁡φF^aφw,φF^aφx=min⁡φF^ψau1,φF^ψau2≤inf⁡φF^ψac:u2∈c  +2  u1=inf⁡φF^aφz,

where *z* ∈ *R*_1_ such that *x* ∈ *z*  +_1_ *w* and *φ*(*z*) = *c*.

Thus it has been proven that $\varphi \left({\hat{F}}_{a}\right)$ is a nonnull subhypergroup of (*R*, +_2_) and consequently $\left(\varphi \left(\hat{F}\right),\psi \left(A\right)\right)$ is a vague soft hypergroup over (*R*, +_2_).

Lastly, let *x*, *y* ∈ *R*_1_. Then there exists *w*_1_, *w*_2_ ∈ *R*_2_ such that *φ*(*x*) = *w*_1_ and *φ*(*y*) = *w*_2_. Therefore for all *x*, *y* ∈ *R*_1_ and $a\in Supp\left(\hat{F},A\right)$ we obtain

(34) min⁡φF^aφx,φF^aφy=min⁡φF^ψaw1,φF^ψaw2≤inf⁡φF^ψap:p∈w1  ∘2  w2=inf⁡φF^aφz,

where *z* ∈ *R*_1_ such that *z* ∈ *x*∘_1_ *y* and *φ*(*z*) = *p*.

Therefore it has been proven that $\varphi \left({\hat{F}}_{a}\right)$ is a nonnull subsemihypergroup of (*R*, ∘_2_) and consequently $\left(\varphi \left(\hat{F}\right),\psi \left(A\right)\right)$ is a vague soft semihypergroup over (*R*, ∘_2_). As such, $\varphi \left({\hat{F}}_{a}\right)$ is a vague subhyperring of (*R*_2_, +_2_, ∘_2_). Hence $\left(\varphi \left(\hat{F}\right),\psi \left(A\right)\right)$ is a vague soft hyperring over (*R*_2_, +_2_, ∘_2_).

**Theorem 49 .**

Let (*R*_1_, +_1_, ∘_1_) and (*R*_2_, +_2_, ∘_2_) be two hyperrings. Let $\left(\hat{F},A\right)$ and $\left(\hat{G},B\right)$ be two vague soft hypergroups over *R*_1_ and *R*_2_, respectively. Let $\left(\varphi ,\psi \right):(\hat{F},A)\to \left(\hat{G},B\right)$ be a vague soft hyperring homomorphism. Then $\left(\varphi^{-1}\left(\hat{G}\right),\psi^{-1}\left(B\right)\right)$ is a vague soft hyperring over (*R*_1_, +_1_, ∘_1_).

**Proof**

The proof is similar to that of Theorem 48.

Theorems 48 and 49 prove that the homomorphic image and preimage of a vague soft hyperring are also a vague soft hyperring. This implies that the vague soft hyperring homomorphism preserves vague soft hyperrings.

**Theorem 50 .**

Let *R*_1_, *R*_2_, and *R*_3_ be nonnull hyperrings and let $\left(\hat{F},A\right)$, $\left(\hat{G},B\right)$, and $\left(\hat{J},C\right)$ be vague soft hyperrings over *R*_1_, *R*_2_, and *R*_3_, respectively. Let (*φ*, *ψ*) : (*F*, *A*) → (*G*, *B*) and (*φ*′, *ψ*′) : (*G*, *B*) → (*J*, *C*) be vague soft hyperring homomorphisms. Then (*φ*′∘*φ*, *ψ*′∘*ψ*) : (*F*, *A*) → (*J*, *C*) is a vague soft hyperring homomorphism.

**Proof**

The proof is straightforward.

## 6. Conclusion

In this paper we introduced and developed the initial theory of vague soft hyperrings as a continuation to the theory of vague soft hypergroups. It was proved that there exists a one-to-one correspondence between the concepts introduced here and the corresponding concepts in soft hyperring theory and classical hyperring theory. Lastly, it was proved that the notion of vague soft hyperring homomorphism introduced here preserves vague soft hyperrings. This is an important step in the formulation and advancement of knowledge in the area of vague soft hyperalgebra.
